# An aeroponic culture system for the study of root herbivory on *Arabidopsis thaliana*

**DOI:** 10.1186/1746-4811-7-5

**Published:** 2011-03-10

**Authors:** Martha M Vaughan, Dorothea Tholl, James G Tokuhisa

**Affiliations:** 1 Department of Biological Sciences, Virginia Tech, Blacksburg, VA 24061, USA; 2 Department of Horticulture, Virginia Tech, Blacksburg, VA 24061, USA; 3 USDA Agricultural Research Service Center for Medical, Agricultural and Veterinary Entomology, Chemistry Research Unit, Gainesville, FL 32608, USA

## Abstract

**Background:**

Plant defense against herbivory has been studied primarily in aerial tissues. However, complex defense mechanisms have evolved in all parts of the plant to combat herbivore attack and these mechanisms are likely to differ in the aerial and subterranean environment. Research investigating defense responses belowground has been hindered by experimental difficulties associated with the accessibility and quality of root tissue and the lack of bioassays using model plants with altered defense profiles.

**Results:**

We have developed an aeroponic culture system based on a calcined clay substrate that allows insect herbivores to feed on plant roots while providing easy recovery of the root tissue. The culture method was validated by a root-herbivore system developed for *Arabidopsis thaliana *and the herbivore *Bradysia *spp. (fungus gnat)*. Arabidopsis *root mass obtained from aeroponically grown plants was comparable to that from other culture systems, and the plants were morphologically normal. *Bradysia *larvae caused considerable root damage resulting in reduced root biomass and water absorption. After feeding on the aeroponically grown root tissue, the larvae pupated and emerged as adults. Root damage of mature plants cultivated in aeroponic substrate was compared to that of *Arabidopsis *seedlings grown in potting mix. Seedlings were notably more susceptible to *Bradysia *feeding than mature plants and showed decreased overall growth and survival rates.

**Conclusions:**

A root-herbivore system consisting of *Arabidopsis thaliana *and larvae of the opportunistic herbivore *Bradysia *spp. has been established that mimics herbivory in the rhizosphere. *Bradysia *infestation of *Arabidopsis *grown in this culture system significantly affects plant performance. The culture method will allow simple profiling and *in vivo *functional analysis of root defenses such as chemical defense metabolites that are released in response to belowground insect attack.

## Background

Belowground herbivory affects plant performance in several ways. For example, insect feeding on plant roots reduces uptake of water and nutrients, limits carbohydrate storage, and changes the production of phytohormones [1-3]. Such alterations in the physical, biochemical, and physiological state of plants can influence surrounding organismal communities both above-and belowground [2,4].

Plants have developed multiple strategies such as tolerance and direct and indirect defenses to cope with or defeat herbivore attack [5-7]. Direct defense mechanisms involve the production of defense proteins and secondary (specialized) plant metabolites, which directly affect herbivores, whereas indirect defenses help attract natural enemies of herbivores [5-11]. In contrast to the many plant defense responses investigated aboveground, fewer studies have focused on plant defenses against root-attacking herbivores [12]. For example, feeding of the cabbage and turnip root maggot (*Delia radicum *and *Delia floralis*) was shown to induce the production of glucosinolate defense metabolites in the roots of several *Brassica *species [13,14]. Schmelz et al. [15] demonstrated that phytoecdysteroids accumulating in spinach roots serve as inducible defense compounds that decrease root feeding of *Bradysia *spp. (fungus gnat) larvae. In addition to direct defenses, some indirect defense responses have been demonstrated belowground. When attacked by larvae of the western corn rootworm (*Diabrotica virgifera virgifera*), maize roots release the sesquiterpene volatile (*E*)-β-caryophyllene, which attracts insect parasitizing nematodes [16].

Detailed investigations of molecular and chemical defense responses in plant roots are limited, which can be largely attributed to experimental shortfalls associated with the accessibility of root tissue or the interference of soil particles with root metabolite analysis [1,17]. Moreover, research on belowground defenses has been hindered by the lack of bioassays using model plants deficient in herbivore-induced root defenses such as the formation of defense metabolites. In response to these challenges, we have developed a root-herbivore system using *Arabidopsis thaliana *and larvae of the root herbivore *Bradysia *spp.

Dark-winged fungus gnats are generalist opportunistic herbivores, whose larvae feed on organic matter and fungi, but upon depletion of this food source, larvae actively feed on root tissue of a variety of plants including *Arabidopsis *[18,19]. Larvae chew on roots and strip away the cortex, thereby negatively impacting water and nutrient absorption [18]. The two most common species, *Bradysia coprophila *(Lintner) and *Bradysia impatiens *(Johannsen), are both considered important greenhouse pests [20], which, at extreme infestations, lead to loss of plant vigor and even mortality [21]. For example, 90% of alfalfa seedlings are killed at densities of less than one larva per seedling [18]. Young soybean plants have been shown to survive *Bradysia coprophila *feeding but produce less seed [18]. These damaging effects of *Bradysia *species can be attributed to their short life cycle of 20 to 25 days with females laying between 250 to 1000 eggs in approximately three days. Emerging larvae feed in the soil for 12 to 14 days prior to pupating [20].

*Bradysia *larvae have been used to investigate hormone-dependent defense responses in *Arabidopsis*. Larvae caused high mortality of soil-grown *Arabidopsis *mutants deficient in the biosynthesis of jasmonic acid, but also affected the growth of wild-type seedlings [19]. Effects on mature plants were described as minor, but this assessment was based only on observations of foliage and survival without investigating the severity of root damage [19]. *Bradysia *feeding was also employed to analyze root-specific defense activities of phytoecdysteroids in spinach [15]. However, this study used *in vitro *feeding assays, instead of an *in vivo *approach, utilizing diets of powdered root tissue containing different phytoecdysteroid concentrations.

In this paper, we describe a culture system based on commercially-available, porous calcined clay pellets (Seramis^®^) to investigate belowground herbivory on *Arabidopsis *roots. The system maintains a soil-type environment for the herbivore while providing easy access to root tissue for analysis. Furthermore, we evaluate feeding by fungus gnat larvae on seedlings grown in soil in comparison to feeding on roots of mature plants in aeroponic culture. We propose this bioassay to be a useful tool for studying chemical- and molecular-defense responses of *Arabidopsis *to belowground herbivory. Moreover, the system has broader utility to investigate many aspects of root biochemistry, physiology, and ecology.

## Methods

### Plant material

*Arabidopsis thaliana *ecotype Columbia (Col-0, ABRC Stock no. 6000) was grown under controlled conditions at 22°C to 25°C, 150 μmol m^-2 ^sec^-1 ^photosynthetically active radiation and a 10 h light - 14 h dark photoperiod. Seeds were stratified for 24 h at 4°C prior to planting in potting mix [90% Sunshine Mix No. 1 (Sun Gro Horticulture, Bellevue, WA) and 10% sand].

### *Arabidopsis *aeroponic culture system

**Figure 1:**
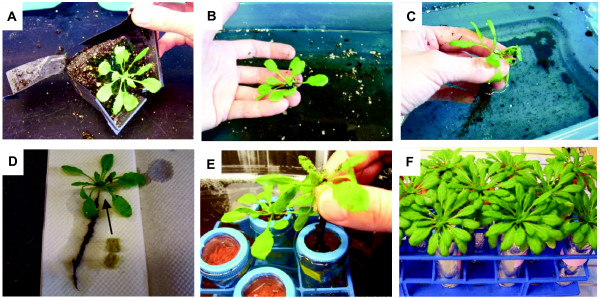
**Technique for growing *Arabidopsis *in aeroponic culture**. (A) Four-week-old *Arabidopsis *plants removed from pots. (B, C) Repeated rinsing of roots in water to remove potting mix. (D) Plants prior to transplant into clay granulate with a small cube of Rockwool (1.25 cm) to be placed around the base of the rosette. (E) Transfer of plants to plastic tubes containing 50 ml of Seramis^® ^clay granules saturated in Hoagland's solution. (F) Plants grown for four weeks under short day conditions following transfer to aeroponic culture tubes. At this stage, plant shoots began to bolt.

Seramis^® ^clay granules http://www.seramis.de are readily available in Europe with currently limited distribution in North America (Ace Gardening Products, Kitchener, Ontario, Canada; http://www.seramis.de/weltweit.html). Fifty ml polypropylene conical tubes (Fisher Scientific, Suwanee, GA) were prepared by drilling five 3.5 mm diameter holes around the bottom and a 25 mm hole in the cap (Figure 1).

Tubes were then filled with Seramis^® ^clay granules and wrapped with aluminum foil to reduce light penetration and algal growth. The top of each tube was covered with plastic wrap and the caps were reattached. A small hole large enough for the root mass to pass through was punctured in the plastic wrap using the tip of a scalpel. The tubes were then placed in a test tube rack and submerged in Hoagland's solution [22] to moisten the substrate. Four-week-old *Arabidopsis *plants grown in potting mix were carefully removed from their pots (Figure 1). Roots were repeatedly submerged in water to remove as much of the potting substrate as possible. A 1.25 cm cube of Rockwool was divided and placed beneath the rosette, around the top of the roots. Plants were then transferred to Seramis^®^-containing tubes and allowed to grow. The Rockwool and plastic wrap stabilized the plant until its roots had grown into the clay granulate. The clay granulate was kept moist by submerging the culture tubes in Hoagland's solution for 10 to 15 min every other day. After four weeks, when the plants started to bolt, roots had grown to the bottom of the tube. During the feeding experiments, plants were watered every other day by applying 10 ml of Hoagland's solution to the surface and allowing excess media to drain freely from the tube.

### *Bradysia *(fungus gnat) culture

Fungus gnat larvae were collected from greenhouse soil by the "potato disk" method [23] and subsequently used to establish a laboratory colony. The colony was maintained in 8 L plastic containers with screened openings for ventilation. The culture medium consisted of 4 L of moist Sunshine Mix No. 1 enriched with 1.5 kg of shredded potato tubers [24]. The cultures were kept at ambient greenhouse conditions (21°C to 23°C; indirect sunlight). To maintain the colony, 0.5 L of medium (containing *Bradysia *larvae, pupae, adults, and eggs) was transferred to a fresh container of soil and potato mix every three to four weeks. Specimens were identified by Dr. Raymond J. Gragné (USDA Systematic Entomology Laboratory, Washington, DC) as a mixed colony of *Bradysia coprophila *(Lintner) and *Bradysia impatiens *(Johannsen).

### Isolation of *Bradysia *larvae

**Figure 2:**
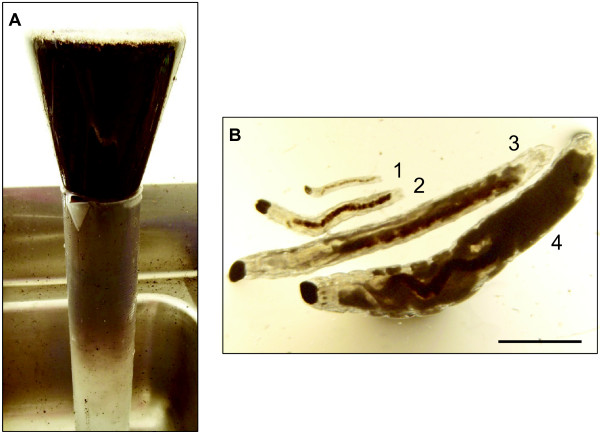
**Floatation method for the collection of *Bradysia *larvae**. (A) A 2 L wide-mouth Erlenmeyer flask inverted on a graduated cylinder and containing fungus gnat larvae and larval culture substrate suspended in tap water. The larvae were collected at the bottom of the cylinder while most of the culture substrate remained floating in the flask. (B) Instars of *Bradysia *larvae collected by this method. Numbers indicate the different instars. Scale bar: 1 mm.

Larvae were collected from the culture using a modified flotation/extraction method previously described by Cloyd and Zaborski [24]. Approximately 1 L of culture medium containing *Bradysia *larvae was transferred to a 2 L wide-mouth Erlenmeyer flask. The flask was filled with room temperature tap water and agitated to break up the culture substrate and release the larvae. The flask was then inverted and placed on top of a 2 L graduated cylinder filled with cold water, so that the mouth of the flask opened just below the water surface in the cylinder (Figure 2A). In this position the flask remained filled with water with the graduated cylinder serving as both a container and a rack to hold the flask. Denser material including fungus gnat larvae sank from the flask into the cylinder while most of the potting mix remained floating in the flask. After approximately 5 min, the flask was removed, and its contents were discarded. Water, larvae, and the remaining organic matter were poured from the graduated cylinder into a plastic container. After the larvae had settled to the bottom of the container, the water was decanted and a 1.5 M MgSO_4 _solution was added. Larvae floated to the top because of their lower density compared to the MgSO_4 _solution, while most of the remaining organic matter sank to the bottom. Larvae were then collected in a sieve (1 mm mesh size), rinsed in water, and placed in a Petri dish with moist filter paper without food for 20 to 24 h prior to their use in feeding assays. The described isolation method collected approximately 1000 *Bradysia *larvae of all four instars from one liter of culture medium (Figure 2B).

### *Bradysia *feeding experiments in aeroponic culture

Four weeks after their transfer to aeroponic culture tubes, *Arabidopsis *plants were infested with fungus gnat larvae. To this end, the plastic tube caps were carefully removed to avoid leaf damage. Approximately 200 to 300 second- and third-instar larvae, collected as described above, were transferred to a single aeroponic culture tube. First instar (~1 mm) and fourth instar larvae were excluded from the feeding experiments since they were either too difficult to apply or too close to pupation. Larvae were submerged in 1 mL of Hoagland's solution (Hoagland's solution was used instead of water to avoid washing nutrients from the clay pellets) and then released with a pipette into the Seramis^® ^substrate. After two and four days of larval feeding, roots were removed from the tubes by submerging the plant and the tube in water. Roots were then separated from the granulate by simply holding the plant rosette and moving it back and forth in the water. The heavier clay granules fell to the bottom of the container, while the roots stayed attached to the rosette. The shoots and roots were then blotted dry with Kimwipes (Fisher Scientific, Suwanee, GA) and their fresh weights were recorded. Tissue dry weight was measured after two to three days of air drying at room temperature. Under lab conditions, this time was sufficient to completely dehydrate the root mass and additional drying did not significantly alter weight measurements. The shoot and root biomass and percentage of water weight for each tissue were calculated. For these measurements a T-test was performed (n = 12, α = 0.05) to test the null hypothesis of no biomass change for the variables. To determine if there was a significant difference in root mass after larval feeding, a one-way analysis of variance (ANOVA) followed by Tukey-Kramer HSD (n = 8) was performed.

### *Bradysia *feeding experiments with seedlings grown in potting mix

To validate the feeding damage by *Bradysia *larvae on roots of aeroponically-grown mature *Arabidopsis *plants, plants were challenged with larval feeding at the seedling stage in potting mix. Individual seedlings were grown in pots (8 cm × 8 cm × 10 cm) under the described conditions. *Bradysia *larvae (second and third instar, Figure 2B), collected as described above, were dislodged from the bottom of the Petri dish with 1 mL of water, and ten larvae were transferred with a Pasteur pipette into each pot. Larvae that did not move into the potting substrate within 5 min were removed and replaced. Larvae were transferred to pots at seed germination, ten and 14 days after germination. Infested and control plants were kept in net enclosures. The percentage of surviving seedlings from three independent experiments was determined seven days after inoculation. Plants were removed from the pots, roots were rinsed in water, and fresh and dry weights of the entire seedling were recorded. For data analysis, the weights were log transformed before performing a one-way ANOVA and Tukey-Kramer HSD (n = 8-10).

### Statistical Analysis

Statistical data analysis was performed as described for the individual experimental procedures. Analysis of variance was accomplished by the JMP program (Version 8, SAS Institute Inc., Cary, NC, USA) statistical software.

## Results

### *Arabidopsis *growth in an aeroponic root culture system

**Figure 3:**
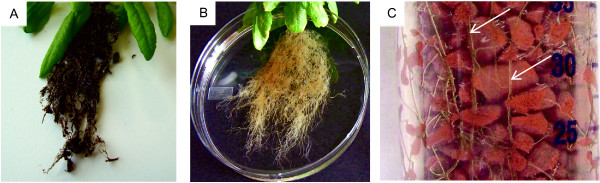
***Arabidopsis *roots in potting substrate and aeroponic culture**. Root morphology of *Arabidopsis *plants grown in potting mix (A) and under aeroponic culture conditions (B). (C) Close-up of roots growing in the aeroponic clay substrate. Arrows indicate root tissue.

**Figure 4:**
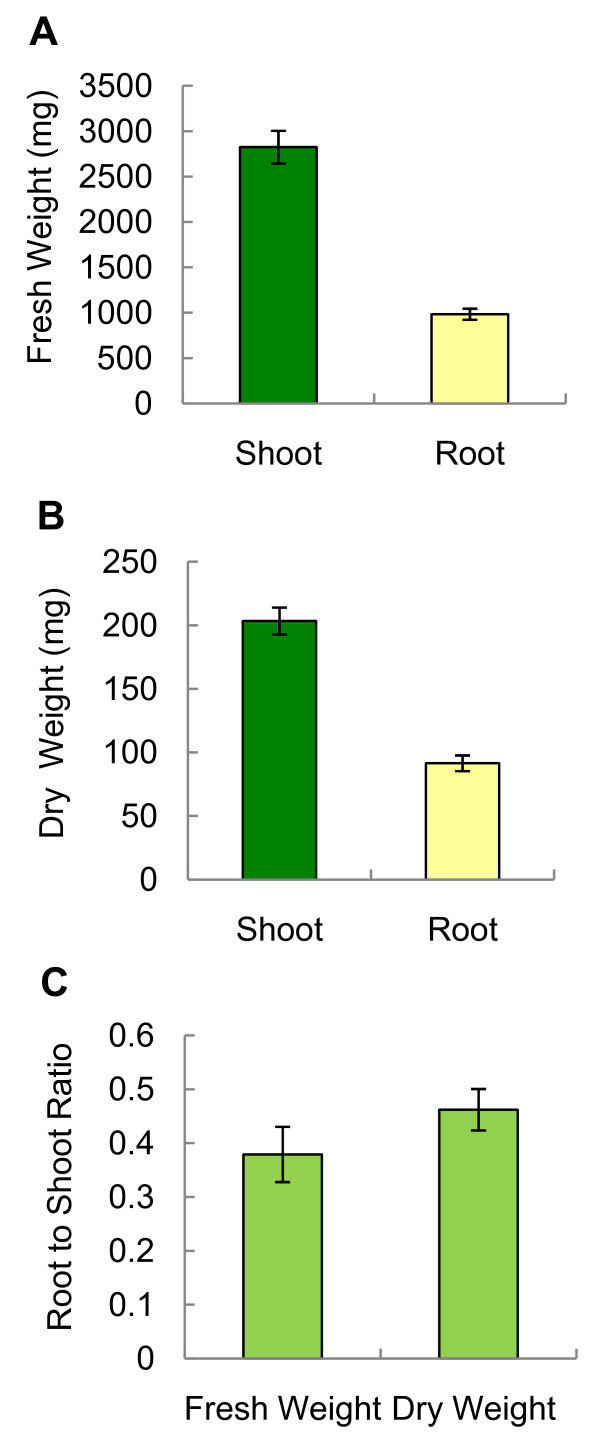
**Biomass analysis of *Arabidopsis *grown in aeroponic culture**. (A) Average shoot and root fresh weight and (B) dry weight are shown. (C) Root-to-shoot ratios of fresh and dry weight. Values represent means ± standard error (n = 12). Shoots include leaves and stems.

To cultivate *Arabidopsis *under aeroponic conditions, plants were first grown on potting mix for approximately four weeks prior to their transfer to aeroponic culture tubes. By four weeks after transition to the clay granulate, roots had grown from the residual potting substrate particles to the bottom of the plastic tube. In comparison to *Arabidopsis *plants grown in potting substrate, aeroponically grown plants appeared morphologically normal in primary and secondary root growth as well as in the formation of root hairs [25] (Figure 3 and see below). The Rockwool was removed without difficulty since roots had not grown into the Rockwool substrate. Approximately 1 g of healthy root tissue was obtained from each individual plant (Table 1). The root-to-shoot ratio for fresh and dry weight was 0.40 and 0.45, respectively (Figure 4C).

**Table 1:** *Arabidopsis thaliana *growth in aeroponic culture (this study) compared to axenic and hydroponic liquid cultures reported in previous studies

		Root		Shoot	
		
	Age (Days)	Fresh weight	Dry Weight	Fresh Weight (mg)	Dry weight
Aeroponic	52(28^a^)	984 ± 61	92 ± 6	2,824 ± 180	203 ± 10
Hydroponic^b^	32	498 ± 57	32 ± 3	1,160 ± 69	121 ± 37
Hydroponic^b^	48	2,916 ± 164	211 ± 10	10,940 ± 499	1,195 ± 37
Axenic^c^	24	1,100	-	-	-

^a ^Number of days in aeroponic culture. ^b^[22]. ^c^[26]

### Larval feeding behavior on aeroponically grown *Arabidopsis *roots

**Figure 5:**
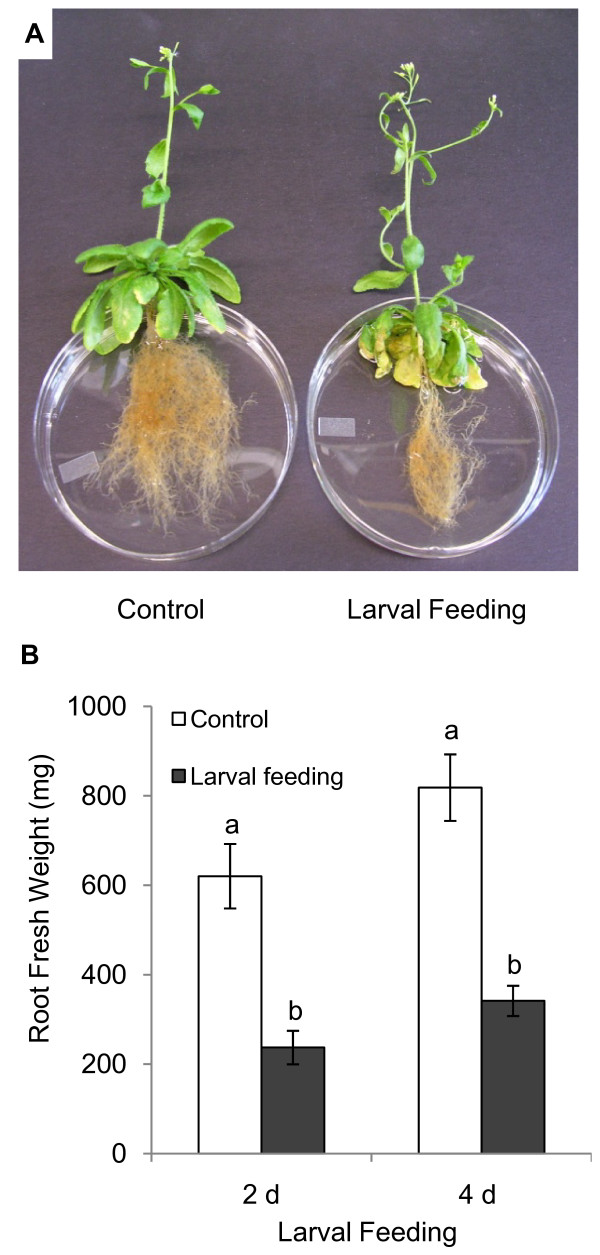
**Feeding damage and root consumption by *Bradysia *larvae on *Arabidopsis *grown in aeroponic culture**. (A) *Arabidopsis *plants removed from clay substrate after four days of larval feeding in comparison to a non-feeding control. Root biomass was visibly reduced and aerial tissues were beginning to wilt. (B) Reduction of root mass after two and four days of herbivory. Values represent averages ± standard error. Letters indicate significant differences between days and treatments (one-way ANOVA and Tukey-Kramer HSD, n = 8, *P *< 0.0001).

**Figure 6:**
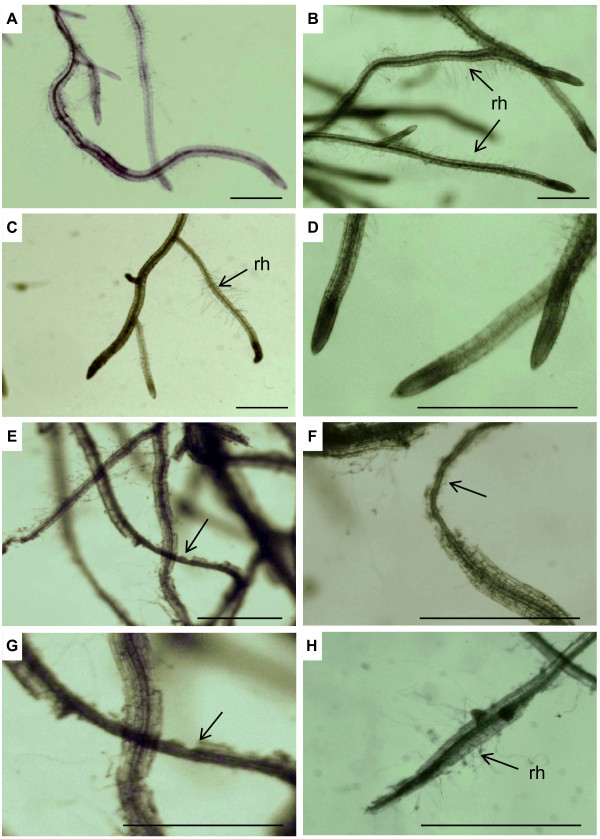
**Roots of aeroponically-grown *Arabidopsis *with and without feeding damage by *Bradysia *larvae**. (A-D) Undamaged roots of eight-week-old *Arabidopsis *grown in aeroponic culture. Primary and secondary roots and root tips (D) are shown. (E-H) Roots damaged by feeding of 200 to 300 *Bradysia *larvae over four days. (E-G) Larvae chewed and stripped away the root epidermis and the cortex but avoided feeding on the vascular tissue (arrows). (H) Feeding damage of root hairs and root tips (rh, root hairs). Scale bar: 500 μm.

Approximately 200 to 300 second and third instar larvae were released into each aeroponic culture tube. Within 24 h, larvae, observed through the clear plastic tube, were actively feeding on the root tissue. Two days after the release of the larvae, severe feeding damage was visible on most roots (Figure 5A and 5B). The majority of root consumption was observed within the upper 5 cm of the tube (Figure 5A) indicating that most larvae fed from the top and gradually moved toward the bottom of the tube as the food source became depleted. Larvae stripped away the root epidermis and cortex, but generally avoided consuming the vascular tissue (Figure 6E-H). Some fine roots were completely severed. Feeding damage was most severe on root tips, young roots, and root hairs suggesting a preference by larvae for these cells and tissues. Thick tap roots displayed little or no feeding damage.

### Effects of *Bradysia *larval feeding on roots of aeroponically grown *Arabidopsis *plants

**Figure 7:**
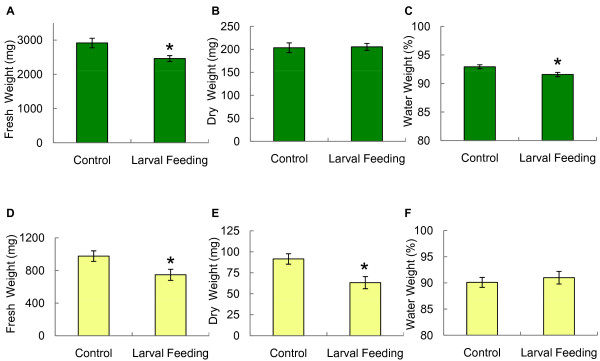
**Shoot and root biomass and water content in response to larval feeding in aeroponic culture**. Shoot fresh weight (A) and dry weight (B) upon four days of larval feeding in comparison to control plants. (C) The percentage of water in the shoot tissue was significantly reduced by root herbivore damage. (D, E) Change in fresh and dry weight of roots in response to larval feeding. (F) The percentage of water weight remained the same. Values represent averages ± standard error. Asterisks above bars indicate significant differences (t-test, n = 12, *P *< 0.02).

Larval root consumption significantly reduced *Arabidopsis *root biomass. On day two and four of larval feeding, the root fresh weight was reduced by 58% and 55%, respectively (Figure 5B). These values reflect the highest reduction of root mass observed in one out of ten independent experiments; the average loss of root mass for all experiments was 37%. In addition to root consumption, stems were poorly supported and rosette leaves were beginning to wilt (Figure 5A). To determine whether leaf wilting was caused by a decreased uptake of water, shoot fresh and dry weight were measured and the weight attributed to water was determined (Figure 7). After four days of larval feeding, shoots showed significantly reduced water content, while shoot dry weight remained unaffected. By contrast, both root fresh and dry weight were significantly reduced with no significant change in the water content of roots due to feeding damage (Figure 7).

### *Bradysia *larval feeding on seedlings grown in potting mix

**Figure 8:**
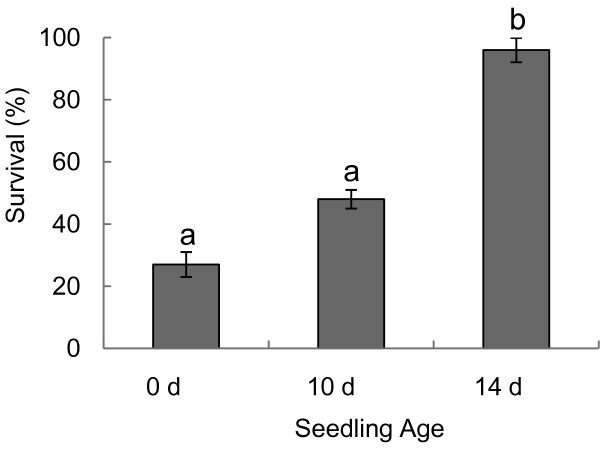
**Survival of *Arabidopsis *seedlings challenged by *Bradysia *larval feeding increased with age**. *Arabidopsis *seedlings were challenged with ten *Bradysia *larvae at germination (0 days), ten days, and 14 days after germination. The average (± standard error) percent of seedling survival was determined from three independent experiments (n = 15) after seven days of larval feeding. Letters indicate significant differences (one-way ANOVA and Tukey-Kramer HSD, *P *< 0.0001).

**Figure 9:**
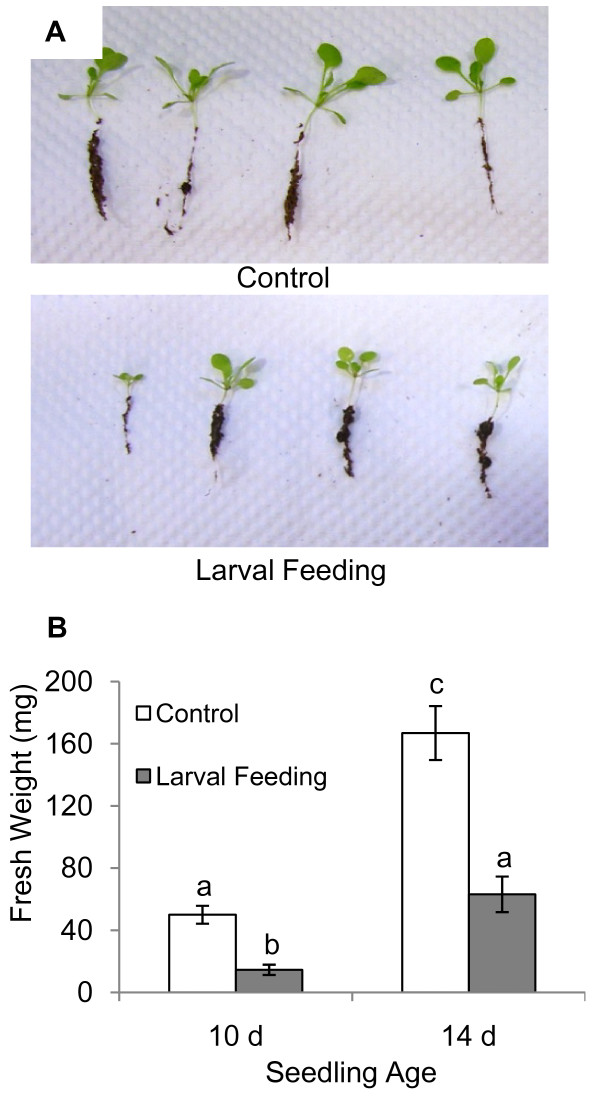
**Effect of *Bradysia *feeding on *Arabidopsis *seedling growth in potting mix**. (A) Developing *Arabidopsis *seedlings challenged by root herbivore feeding were noticeably stunted in growth. (B) Ten and 14-day-old *Arabidopsis *seedlings treated with ten *Bradysia *larvae were significantly reduced in plant biomass. Values represent averages ± standard error. Letters indicate significant differences between seedling ages and treatments (one-way ANOVA and Tukey-Kramer HSD on log(x) transformed data, n = 8-10, *P *< 0.0001).

*Bradysia *larvae reduced *Arabidopsis *seedling establishment. Only 24 to 33% of seedlings survived when exposed to ten *Bradysia *larvae at germination (Figure 8). When 10-day-old seedlings were treated with larvae, survival rates increased to 45 and 51%. However, more than 95% of seedlings survived when larval treatment started after the first pair of true leaves had developed ten to 14 days after germination. Nevertheless, all of the surviving plants were considerably affected by herbivore damage since they had significantly reduced biomass in comparison to unchallenged seedlings and were noticeably stunted in growth (Figure 9).

## Discussion

### *Arabidopsis *aeroponic root cultures provide conditions suitable for root herbivory

Investigating root herbivory has remained a challenge in the study of belowground plant-organism interactions because of limited access to root tissue for subsequent analyses. Several culture methods have been developed previously to make *Arabidopsis *roots more accessible for molecular and biochemical studies. These methods are primarily based on growing plants in liquid culture for optimal root biomass production, but they are not suitable for investigating root herbivory [22,26]. We tested whether hydroponically grown roots removed from liquid culture could be used for *Bradysia *feeding experiments; however, larvae did not actively feed on these roots. Similarly, larvae did not feed on roots of plants grown in solid sterile media, but instead burrowed into the agar and suffocated. *Arabidopsis *hairy root cultures have been previously used to investigate root-aphid interactions and the emission of volatile organic compounds in response to aphid attack [27]. However, in this study aphid feeding was confined to floating root tissue, which simulates the natural habitat of the herbivore only marginally and prevents analyzing responses of the whole plant.

To study herbivory on *Arabidopsis *roots, it was necessary to establish a culture system that could accommodate belowground herbivores while still providing easy access to the root tissue for further molecular or biochemical analysis. Initial attempts to grow *Arabidopsis *in sand and vermiculite resulted in poor plant growth in comparison to soilless potting mix. By contrast, optimal growth conditions were achieved by growing plants in nonsterile perlite or Seramis^® ^clay granules. Seramis^® ^substrate was preferred over perlite since roots could easily be removed from the clay granules by submersion in water without causing any substantial tissue damage.

Optimal survival of plants was achieved when plants were first grown on potting mix for approximately 4 weeks prior to their transfer to aeroponic culture tubes. Transfer of younger plants was avoided due to inconsistent growth performance under aeroponic conditions. Growing seedlings directly on Rockwool placed on top of the clay substrate is less suitable since plants are easily stressed by over- or under-watering. A similar problem has been encountered in establishing hydroponic cultures and is considered a major limitation in the success of germination and seedling survival in this system [22,26]. Within two to three days after transplanting, plants adapt to the aeroponic conditions and grow as phenotypically healthy mature plants with no obvious signs of impaired root growth or damage after 4 weeks (Figure 3 and 6A-D). The culture requires eight weeks to progress from seed to mature plants, but beyond transferring the plants from soil to aeroponic substrate, only watering with Hoagland's solution is necessary. The harvest of roots from the aeroponic system is rapid, complete, and causes negligible damage to roots (Figure 5A and 6A-D) in contrast to removal of potting mix (Figure 1B-D and 9).

Aeroponically-grown plants produce a root mass comparable to that obtained in other previously reported *Arabidopsis *culture systems (Table 1). For example, plants grown for 24 days in axenic liquid culture supplemented with 1 to 3% sucrose yield approximately 1 g of root fresh weight [26], which is similar to that of roots grown in the aeroponic system (Table 1). When compared to *Arabidopsis *grown under hydroponic conditions, the average root-to-shoot ratio in aeroponic culture (0.45) is approximately twice the ratio observed for hydroponically-grown plants [22]. Thirty percent of the total aeroponic plant biomass (dry weight) is comprised of root tissue while only 15 to 25% of total hydroponic plant biomass has been attributed to root tissue [22] (Table 1). The higher percentage of root mass under aeroponic conditions may be the result of increased aeration in the root environment [28,29]. Moreover, enhanced root growth might be caused by the intermittent nutrient supply in aeroponic culture as opposed to continuous nutrient availability in hydroponic systems.

Taken together, the technique shown here represents a simple culture method, which can be established under ordinary growth conditions without the use of sophisticated hydroponic equipment such as bubble stones, air pumps or sprayers. In comparison to hydroponic cultivation, the culture system reflects more closely the conditions of the soil environment. Since clay granules such as Seramis^® ^are used as a common plant medium in home gardens and interior landscapes http://www.seramis.de, aeroponic cultures similar to the one presented here can be established for a variety of different plants including those with a longer life span than *Arabidopsis*. Because of the loosely packed consistency of the clay substrate, it seems possible that root herbivores of larger size than *Bradysia *larvae can be accommodated in this substrate, which will allow testing this method for other root-herbivore systems.

### *Bradysia *larval feeding significantly reduces root biomass of mature *Arabidopsis *plants and affects survival and growth of seedlings

Fungus gnats are common greenhouse pests and frequently infest *Arabidopsis *growth rooms. While feeding of *Bradysia *larvae on *Arabidopsis *seedlings has been previously reported [19], the extent of feeding damage on mature wild-type plants has not been investigated. When placed in aeroponic clay substrate deprived of other food sources, larvae quickly began to feed on root tissue. Within two to four days of feeding, larvae significantly reduced root mass by an average of 37%. Despite the considerable damage of tissue belowground, plants appeared to be less affected aboveground. However, *Bradysia*-damaged plants showed reduced shoot mass, which was due to the loss of water, suggesting that water absorption and most likely nutrient absorption were compromised by root herbivore damage.

In the presented experiments, approximately 200 to 300 larvae at the second and third instar were applied per plant. Considering that only 20 to 25 gnats released into an area with 15 pots containing *Arabidopsis *plants can yield 300 to 500 larvae per pot within 14 days [19], it was within reason that this density can occur under greenhouse conditions. The observed root damage in aeroponic culture may be magnified due to the absence of other food sources such as organic matter. *Bradysia *larvae primarily consumed root tips, root hairs, and young lateral roots (Figure 6E-H). Less feeding was observed on older roots, where larvae preferably removed the epidermis and cortex but none of the vascular tissue and only parts of the endodermis. This feeding preference may be caused in part by increased root secondary growth. An increase in cell wall lignification can affect root herbivore activity as shown for wireworm feeding on tobacco roots [30]. Moreover, differences in formation and concentration of constitutive and induced secondary defense metabolites such as glucosinolates and terpenoids can contribute to the observed differential feeding behavior and overall root consumption (Vaughan, Tokuhisa, and Tholl, unpublished results). The role of direct defense metabolites was also demonstrated in spinach roots, where *Bradysia *larvae led to a dramatic up-regulation of defense compounds [15]. A recent study investigating glucosinolate distributions in canola (*Brassica napus*) roots showed that the highest concentration of glucosinolates occurs in the outer layer of roots with secondary growth [31,32]. To what extent *Bradysia *feeding is affected by the distribution of glucosinolates in *Arabidopsis *roots requires further investigation.

Depending on the developmental stage, *Arabidopsis *seedlings grown in potting mix were severely affected by *Bradysia *larval feeding. A moderate infestation [18] of ten larvae per plant reduced seedling survival by 50% or less depending on the plant age at the time the biotic stress was introduced. Surviving seedlings were much smaller and appeared to have reduced growth. This reduction in plant growth can be attributed to larval root consumption and limited uptake of nutrients but may also reflect fundamental changes in the physiological state of the plant due to trade-offs between resource allocation to defense and growth [33,34] or changes in the potting mix due to frass deposition.

To our knowledge, a bioassay for studying belowground herbivory on intact *Arabidopsis *plants has not previously been reported. We propose that *Arabidopsis *grown in aeroponic culture and the generalist herbivore *Bradysia *(*B. coprophila *and *B. impatiens*) can be used as a novel system to investigate belowground plant defense responses to herbivore attack such as the release of secondary metabolites. The chemistry of plant defense against root herbivores is one of the most neglected aspects of root biology [12]. Using this system, *Arabidopsis *mutants with altered profiles of secondary metabolites, such as glucosinolates [35] or terpenoids, can be applied to study the biological effect of individual defense compounds belowground. Finally, knowledge gained from these and various other mutants may provide closer insight into the still poorly understood molecular and physiological mechanisms of belowground and aboveground interactions [36].

## Conclusions

We have established a method of growing *Arabidopsis *in a clay granulate culture system, which can be used to investigate root biology including belowground herbivory and allows for easy preparation of roots for chemical and molecular analyses upon insect feeding. We have shown that *Bradysia *larvae actively feed on mature *Arabidopsis *in aeroponic culture and cause severe root damage that affects both root biomass and water absorption. Furthermore, *Arabidopsis *seedlings challenged by *Bradysia *larvae under conventional cultivation show decreased survival and growth. This root-herbivore system can be used to study the biochemistry, molecular regulation, and function of root defense compounds in response to belowground herbivory.

## Competing interests

The authors declare that they have no competing interests.

## Authors' contributions

MMV carried out all experiments and drafted the manuscript. JGT contributed to intellectual conception and design of the method, helped with drafting the manuscript, and edited the manuscript. DT participated in the study design and coordination, helped with drafting the manuscript, and edited the manuscript. All authors read and approved the final version of the manuscript.
